# Supplementary material for: DMRforPairs: identifying Differentially Methylated Regions between unique samples using array based methylation profiles
Source: BMC Bioinformatics. 2014 May 15;15:141. doi: 10.1186/1471-2105-15-141 (PMC4046028; doi:10.1186/1471-2105-15-141)
Supplement: Additional file 2 — DMRforPairs output for the comparison of A431-MCF7 and NA17018-NA17105. Please start from the HTML files in each folder. Available via the BMC Bioinformatics website. [file 1471-2105-15-141-S2.zip › 1394847754114233_MOESM2_ESM/A431_MCF7/figures/37521.pdf]

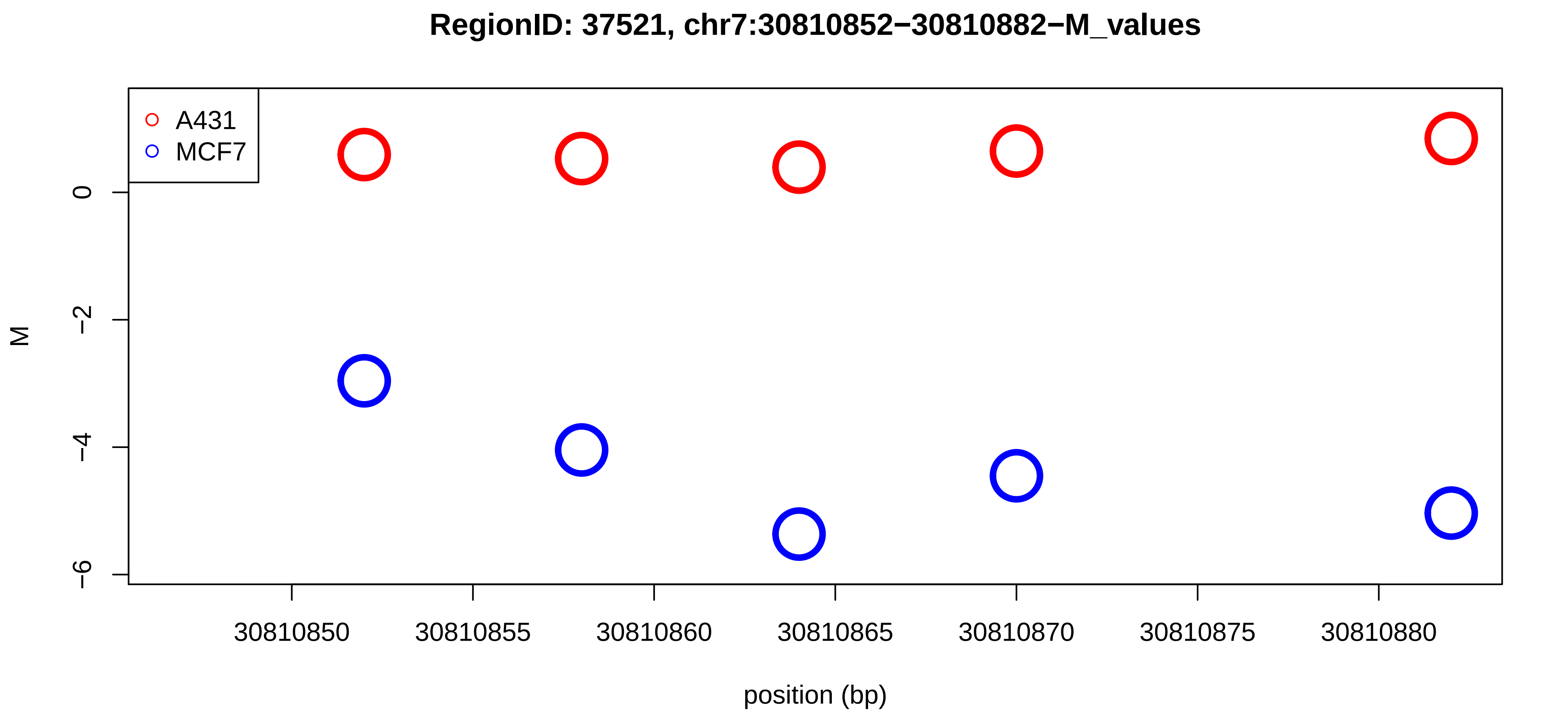
RegionID: 37521, chr7:30810852−30810882−M_values
A431
MCF7
M
0
−2
−4
−6
30810850
30810855
30810860
30810865
30810870
30810875
30810880
position (bp)

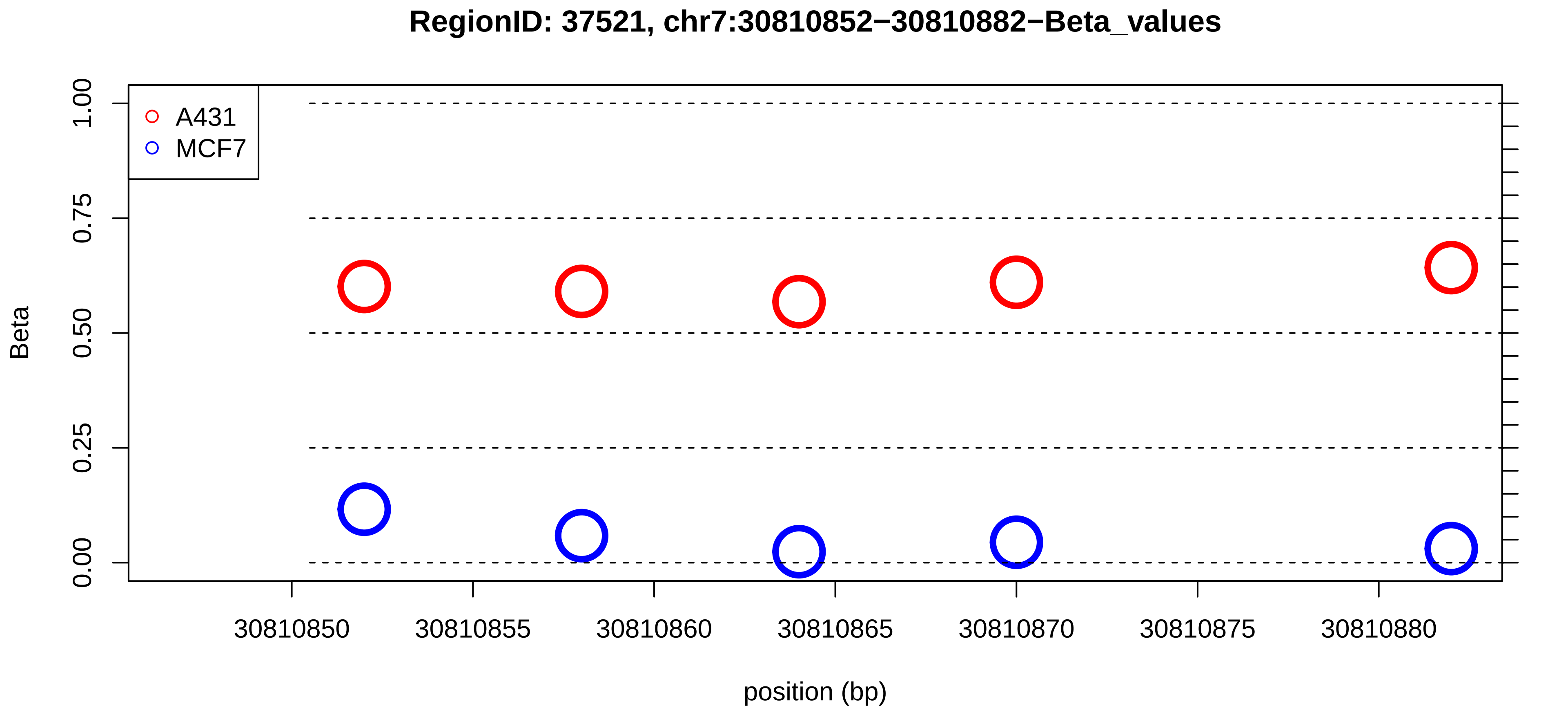
RegionID: 37521, chr7:30810852−30810882−Beta_values
A431
MCF7
Beta
1.00
0.75
0.50
0.25
0.00
30810850
30810855
30810860
30810865
30810870
30810875
30810880
position (bp)

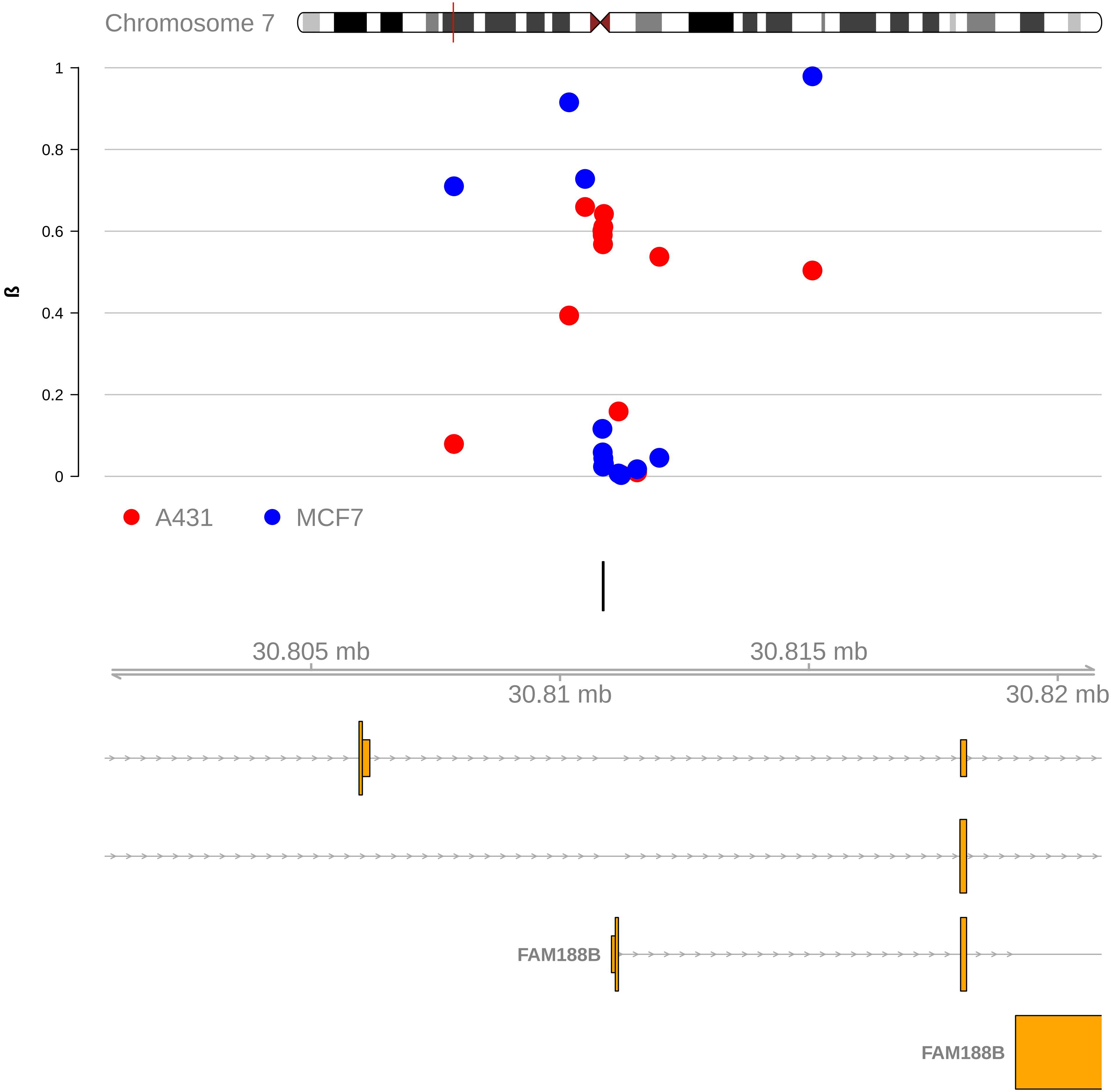
Chromosome 7
1
0.8
0.6
0.4
0.2
0
ß
A431
MCF7
30.805 mb
30.815 mb
30.81 mb
30.82 mb
FAM188B
FAM188B
